# Ethyl 2-[(2,6-dimethyl­phen­yl)hydrazono]-3-oxobutanoate

**DOI:** 10.1107/S160053680902131X

**Published:** 2009-06-10

**Authors:** Hoong-Kun Fun, Samuel Robinson Jebas, Mahesh Padaki, Chitrakar Hegde, Arun M Isloor

**Affiliations:** a X-ray Crystallography Unit, School of Physics, Universiti Sains Malaysia, 11800 USM, Penang, Malaysia; b Department of Chemistry, National Institute of Technology-Karnataka, Surathkal, Mangalore 575 025, India; c Department of Chemistry, NITTE Meenakshi Institute of Technology, Yelahanka, Bangalore 64, India

## Abstract

The title compound, C_14_H_18_N_2_O_3_, crystallizes with two independent mol­ecules in the asymmetric unit, having closely comparable geometries. Both mol­ecules are essentially planar [maximum deviations from the mean plane of 0.069 (1) and 0.068 (1) Å for the two mol­ecules] and contain an intra­molecular N—H⋯O hydrogen bond which generates a ring with graph-set motif *S*(6). In the crystal, the mol­ecules are linked into chains along the *c* axis by inter­molecular C—H⋯O hydrogen bonds, and inter­molecular C—H⋯π inter­actions are also present.

## Related literature

For details of the isolation and cytotoxic properties of oxobutanoate derivatives, see: Billington *et al.* (1979 ▶); Stanchev *et al.* (2008 ▶). For related structures, see: Alpaslan *et al.* (2005 ▶); Fun *et al.* (2009 ▶). For details of the synthesis, see: Amir & Agarwal, (1997 ▶). For the stability of the temperature controller used in the data collection, see: Cosier & Glazer (1986 ▶).
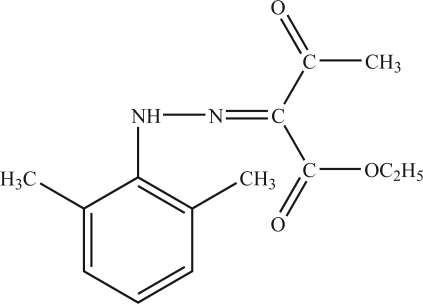


## Experimental

### 

#### Crystal data

C_14_H_18_N_2_O_3_
*M* *_r_* = 262.30
Monoclinic, 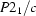

*a* = 6.8072 (1) Å
*b* = 17.4339 (2) Å
*c* = 22.9238 (3) Å
β = 90.921 (1)°
*V* = 2720.15 (6) Å^3^
*Z* = 8
Mo *K*α radiation
μ = 0.09 mm^−1^
*T* = 100 K
0.43 × 0.22 × 0.10 mm

#### Data collection

Bruker SMART APEXII CCD diffractometer
Absorption correction: multi-scan (*SADABS*; Bruker, 2005 ▶) *T*_min_ = 0.962, *T*_max_ = 0.991
38064 measured reflections
6206 independent reflections
4862 reflections with *I* > 2σ(*I*)
*R*_int_ = 0.037

#### Refinement

*R*[*F* ^2^ > 2σ(*F* ^2^)] = 0.043
*wR*(*F* ^2^) = 0.110
*S* = 1.03
6206 reflections
359 parameters
H atoms treated by a mixture of independent and constrained refinement
Δρ_max_ = 0.29 e Å^−3^
Δρ_min_ = −0.27 e Å^−3^

### 

Data collection: *APEX2* (Bruker, 2005 ▶); cell refinement: *SAINT* (Bruker, 2005 ▶); data reduction: *SAINT* program(s) used to solve structure: *SHELXTL* (Sheldrick, 2008 ▶); program(s) used to refine structure: *SHELXTL*; molecular graphics: *SHELXTL*; software used to prepare material for publication: *SHELXTL* and *PLATON* (Spek, 2009 ▶).

## Supplementary Material

Crystal structure: contains datablocks global, I. DOI: 10.1107/S160053680902131X/bi2373sup1.cif

Structure factors: contains datablocks I. DOI: 10.1107/S160053680902131X/bi2373Isup2.hkl

Additional supplementary materials:  crystallographic information; 3D view; checkCIF report

## Figures and Tables

**Table 1:** Hydrogen-bond geometry (Å, °)

*D*—H⋯*A*	*D*—H	H⋯*A*	*D*⋯*A*	*D*—H⋯*A*
C2*B*—H2*BA*⋯O2*B*^i^	0.93	2.54	3.2442 (16)	133
N1*A*—H1*NA*⋯O1*A*	0.904 (18)	1.790 (18)	2.5366 (14)	138.3 (15)
N1*B*—H1*NB*⋯O1*B*	0.91 (2)	1.81 (2)	2.5512 (15)	136.2 (16)
C11*B*—H11*C*⋯*Cg*1^ii^	0.96	2.68	3.5516 (15)	150
C11*B*—H11*D*⋯*Cg*1^iii^	0.96	2.60	3.4832 (15)	152
C11*A*—H11*A*⋯*Cg*2^iv^	0.96	2.61	3.4633 (15)	147
C11*A*—H11*B*⋯*Cg*2^v^	0.96	2.65	3.5304 (15)	151

Symmetry codes: (i) 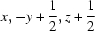; (ii) 
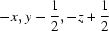
; (iii) 
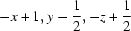
; (iv) 
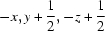
; (v) 
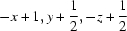
. *Cg*1 and *Cg*2 are the centroids of the C1*A*–C6*A* and C1*B*–C6*B* rings, respectively.

**Fig. 1.**
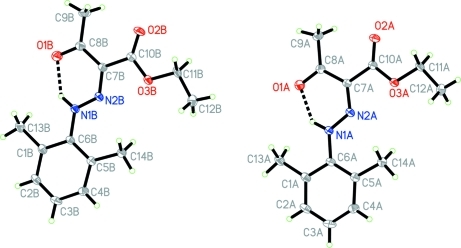
Molecular structure of the title compound, showing 50% probability displacement ellipsoids for non-H atoms

**Fig. 2.**
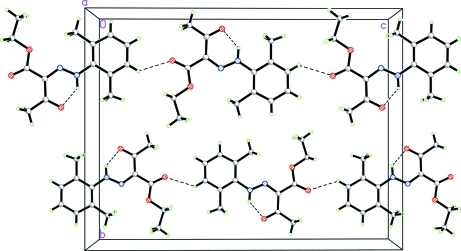
Packing diagram viewed along the a axis, showing molecular chains along the c axis. Dashed lines indicate hydrogen bonds.

## supplementary crystallographic information

### Comment

Derivatives of oxobutanoates are biologically important due to their interesting properties. 4-Methylthio-2-oxobutanoate was identified in the culture fluids of a range of bacteria, the yeast *Saccharomyces cerevisiae* and the fungus *Penicillium digitatum* (Billington *et al.*, 1979). Some of the oxobutanoate exhibited cytotoxic property (Stanchev *et al.*, 2008). Crystal structures of ethyl 4-chloro-2-[2-(2-methoxyphenyl)hydrazono]-3-oxobutanoate (Alpaslan *et al.*, 2005) and ethyl 2-[(4-chlorophenyl)hydrazono]-3-oxobutanoate (Fun *et al.*, 2009) have been reported.

There are two independent molecules (*A* and *B*) in the asymmetric unit of the title compound (Fig. 1). The benzene rings in the two molecules (C1A–C6A and C1B—C6B) are almost coplanar, forming a dihedral angle of 3.14 (6)°. The mean plane of the part of the oxobutanoate unit, C7A—C12A/O3A in molecule *A* and C7B—C12B/O3B in molecule *B*, is slightly twisted from the mean planes of the phenyl rings, (C1A—C6A) and (C1B—C6B), forming dihedral angles of 2.26 (7)° in molecule *A* and 2.16 (8)° in molecule *B* respectively. An intramolecular N—H···O hydrogen bond is present in both molecules, generating a ring with graph-set motif S(6).

In the crystal packing (Fig. 2), the molecules are linked into chains along the *c* axis by intermolecular C—H···O hydrogen bonds. Intramolecular O···N short contacts (2.5362 (13)Å and 2.8224 (14)Å) and intermolecular C—H···π interactions (Table 1) are also observed.

### Experimental

The title compound was prepared according to a literature procedure (Amir & Agarwal, 1997): 2,6-dimethylaniline (2.26 g, 0.01 mol) was dissolved in dilute hydrochloric acid (22.0 ml, 9.0 ml HCl dissolved in 13.0 ml water) and cooled to 0°C in an ice bath. To this, a cold solution of sodium nitrite (3.2 g, 0.0462 mol in 10.0 ml water) was added, with the temperature of the reaction mixture kept below 5°C. The resulting diazonium salt solution was filtered into a cooled solution of ethylacetoacetate (3.4 ml) and sodium acetate (7.0 g) in ethanol (100 ml). The resulting yellow-orange solid was filtered, washed with ice cold water, dried in air and recrystallized from methanol. Yield 3.65 g (86.5%), m.p. 338–340 K.

### Refinement

H atoms were positioned geometrically [C–H = 0.93–0.97 Å] and refined using a riding model, with *U*_iso_(H) = 1.2*U*_eq_(C) and 1.5*U*_eq_(methyl C). A rotating-group model was used for the methyl groups. H atoms bound to N were located from a Fourier map and allowed to refine freely.

### Crystal data

**Table:**

C_14_H_18_N_2_O_3_	*F*(000) = 1120
*M**_r_* = 262.30	*D*_x_ = 1.281 Mg m^−^^3^
Monoclinic, *P*2_1_/*c*	Mo *K*α radiation, λ = 0.71073 Å
Hall symbol: -P 2ybc	Cell parameters from 9924 reflections
*a* = 6.8072 (1) Å	θ = 2.9–33.1°
*b* = 17.4339 (2) Å	µ = 0.09 mm^−^^1^
*c* = 22.9238 (3) Å	*T* = 100 K
β = 90.921 (1)°	Plate, yellow
*V* = 2720.15 (6) Å^3^	0.43 × 0.22 × 0.10 mm
*Z* = 8	

### Data collection

**Table:**

Bruker SMART APEXII CCD diffractometer	6206 independent reflections
Radiation source: fine-focus sealed tube	4862 reflections with *I* > 2σ(*I*)
graphite	*R*_int_ = 0.037
φ and ω scans	θ_max_ = 27.5°, θ_min_ = 2.1°
Absorption correction: multi-scan (*SADABS*; Bruker, 2005)	*h* = −8→8
*T*_min_ = 0.962, *T*_max_ = 0.991	*k* = −22→22
38064 measured reflections	*l* = −29→29

### Refinement

**Table:**

Refinement on *F*^2^	Primary atom site location: structure-invariant direct methods
Least-squares matrix: full	Secondary atom site location: difference Fourier map
*R*[*F*^2^ > 2σ(*F*^2^)] = 0.043	Hydrogen site location: inferred from neighbouring sites
*wR*(*F*^2^) = 0.110	H atoms treated by a mixture of independent and constrained refinement
*S* = 1.03	*w* = 1/[σ^2^(*F*_o_^2^) + (0.0474*P*)^2^ + 1.0468*P*] where *P* = (*F*_o_^2^ + 2*F*_c_^2^)/3
6206 reflections	(Δ/σ)_max_ < 0.001
359 parameters	Δρ_max_ = 0.29 e Å^−^^3^
0 restraints	Δρ_min_ = −0.27 e Å^−^^3^

### Special details

**Table:**

Experimental. The crystal was placed in the cold stream of an Oxford Cyrosystems Cobra open-flow nitrogen cryostat operating at 110.0 (1) K (Cosier & Glazer, 1986).
Geometry. All e.s.d.'s (except the e.s.d. in the dihedral angle between two l.s. planes) are estimated using the full covariance matrix. The cell e.s.d.'s are taken into account individually in the estimation of e.s.d.'s in distances, angles and torsion angles; correlations between e.s.d.'s in cell parameters are only used when they are defined by crystal symmetry. An approximate (isotropic) treatment of cell e.s.d.'s is used for estimating e.s.d.'s involving l.s. planes.
Refinement. Refinement of *F*^2^ against ALL reflections. The weighted *R*-factor *wR* and goodness of fit *S* are based on *F*^2^, conventional *R*-factors *R* are based on *F*, with *F* set to zero for negative *F*^2^. The threshold expression of *F*^2^ > σ(*F*^2^) is used only for calculating *R*-factors(gt) *etc*. and is not relevant to the choice of reflections for refinement. *R*-factors based on *F*^2^ are statistically about twice as large as those based on *F*, and *R*- factors based on ALL data will be even larger.

### Fractional atomic coordinates and isotropic or equivalent isotropic displacement parameters (Å^2^)

**Table:**

	*x*	*y*	*z*	*U*_iso_*/*U*_eq_	
O1A	0.40917 (16)	0.60365 (5)	0.14873 (4)	0.0254 (2)	
O2A	0.40976 (16)	0.67847 (5)	−0.02578 (4)	0.0247 (2)	
O3A	0.40376 (15)	0.79917 (5)	0.00793 (4)	0.0184 (2)	
N1A	0.40541 (17)	0.74634 (6)	0.17034 (4)	0.0154 (2)	
N2A	0.40625 (16)	0.75908 (6)	0.11429 (4)	0.0148 (2)	
C1A	0.4144 (2)	0.77921 (8)	0.27072 (5)	0.0181 (3)	
C2A	0.4216 (2)	0.83300 (8)	0.31550 (6)	0.0225 (3)	
H2AA	0.4252	0.8166	0.3541	0.027*	
C3A	0.4235 (2)	0.91073 (8)	0.30312 (6)	0.0236 (3)	
H3AA	0.4306	0.9463	0.3333	0.028*	
C4A	0.4149 (2)	0.93537 (8)	0.24572 (6)	0.0200 (3)	
H4AA	0.4145	0.9878	0.2381	0.024*	
C5A	0.40668 (19)	0.88417 (8)	0.19881 (5)	0.0160 (3)	
C6A	0.40846 (19)	0.80554 (8)	0.21245 (5)	0.0154 (3)	
C7A	0.4061 (2)	0.70016 (7)	0.07753 (5)	0.0150 (3)	
C8A	0.4068 (2)	0.61935 (8)	0.09568 (6)	0.0182 (3)	
C9A	0.4072 (2)	0.55506 (8)	0.05250 (6)	0.0255 (3)	
H9AA	0.4085	0.5069	0.0729	0.038*	
H9AB	0.5218	0.5588	0.0288	0.038*	
H9AC	0.2915	0.5582	0.0281	0.038*	
C10A	0.4071 (2)	0.72277 (8)	0.01496 (5)	0.0155 (3)	
C11A	0.4007 (2)	0.82543 (8)	−0.05216 (5)	0.0175 (3)	
H11A	0.2858	0.8056	−0.0727	0.021*	
H11B	0.5172	0.8080	−0.0721	0.021*	
C12A	0.3949 (2)	0.91184 (8)	−0.05037 (6)	0.0216 (3)	
H12A	0.3835	0.9315	−0.0894	0.032*	
H12B	0.5136	0.9308	−0.0323	0.032*	
H12C	0.2840	0.9283	−0.0282	0.032*	
C13A	0.4136 (2)	0.69449 (8)	0.28431 (6)	0.0241 (3)	
H13A	0.4161	0.6872	0.3258	0.036*	
H13B	0.5272	0.6708	0.2678	0.036*	
H13C	0.2969	0.6715	0.2680	0.036*	
C14A	0.3951 (2)	0.91598 (8)	0.13753 (6)	0.0201 (3)	
H14A	0.3896	0.9710	0.1391	0.030*	
H14B	0.2792	0.8968	0.1181	0.030*	
H14C	0.5092	0.9004	0.1165	0.030*	
O1B	0.09517 (17)	0.08911 (5)	0.43092 (4)	0.0262 (2)	
O2B	0.09218 (17)	0.21913 (6)	0.27668 (4)	0.0263 (2)	
O3B	0.08746 (15)	0.32485 (5)	0.33244 (4)	0.0189 (2)	
N1B	0.09558 (17)	0.22067 (7)	0.47965 (4)	0.0153 (2)	
N2B	0.09259 (16)	0.25040 (6)	0.42748 (4)	0.0147 (2)	
C1B	0.0930 (2)	0.22404 (8)	0.58338 (5)	0.0179 (3)	
C2B	0.0920 (2)	0.26448 (8)	0.63570 (6)	0.0209 (3)	
H2BA	0.0895	0.2377	0.6708	0.025*	
C3B	0.0948 (2)	0.34375 (8)	0.63647 (6)	0.0214 (3)	
H3BA	0.0938	0.3700	0.6718	0.026*	
C4B	0.0992 (2)	0.38391 (8)	0.58425 (6)	0.0180 (3)	
H4BA	0.1014	0.4372	0.5853	0.022*	
C5B	0.10050 (19)	0.34683 (7)	0.53016 (5)	0.0151 (3)	
C6B	0.09607 (19)	0.26601 (7)	0.53072 (5)	0.0144 (3)	
C7B	0.0909 (2)	0.20567 (7)	0.38054 (5)	0.0153 (3)	
C8B	0.0895 (2)	0.12139 (8)	0.38275 (6)	0.0183 (3)	
C9B	0.0792 (2)	0.07339 (8)	0.32851 (6)	0.0256 (3)	
H9BA	0.0734	0.0201	0.3390	0.038*	
H9BB	0.1938	0.0825	0.3056	0.038*	
H9BC	−0.0362	0.0868	0.3062	0.038*	
C10B	0.0897 (2)	0.24863 (8)	0.32454 (5)	0.0166 (3)	
C11B	0.0923 (2)	0.37110 (8)	0.27969 (5)	0.0186 (3)	
H11C	−0.0236	0.3615	0.2556	0.022*	
H11D	0.2077	0.3589	0.2573	0.022*	
C12B	0.0979 (2)	0.45359 (8)	0.29895 (6)	0.0229 (3)	
H12D	0.1118	0.4862	0.2655	0.034*	
H12E	0.2073	0.4613	0.3252	0.034*	
H12F	−0.0219	0.4660	0.3184	0.034*	
C13B	0.0906 (2)	0.13761 (8)	0.58360 (6)	0.0260 (3)	
H13D	0.0922	0.1194	0.6231	0.039*	
H13E	0.2042	0.1187	0.5639	0.039*	
H13F	−0.0261	0.1197	0.5639	0.039*	
C14B	0.1078 (2)	0.39513 (8)	0.47569 (6)	0.0192 (3)	
H14D	0.1133	0.4484	0.4862	0.029*	
H14E	−0.0076	0.3858	0.4522	0.029*	
H14F	0.2225	0.3820	0.4540	0.029*	
H1NA	0.403 (3)	0.6967 (11)	0.1816 (7)	0.037 (5)*	
H1NB	0.099 (3)	0.1685 (12)	0.4821 (8)	0.048 (6)*	

### Atomic displacement parameters (Å^2^)

**Table:**

	*U*^11^	*U*^22^	*U*^33^	*U*^12^	*U*^13^	*U*^23^
O1A	0.0396 (7)	0.0191 (5)	0.0174 (5)	−0.0012 (5)	0.0000 (4)	0.0023 (4)
O2A	0.0393 (7)	0.0208 (5)	0.0139 (4)	−0.0009 (5)	0.0021 (4)	−0.0042 (4)
O3A	0.0259 (6)	0.0170 (5)	0.0122 (4)	−0.0002 (4)	0.0006 (4)	0.0001 (3)
N1A	0.0180 (6)	0.0166 (6)	0.0115 (5)	−0.0003 (5)	0.0002 (4)	−0.0003 (4)
N2A	0.0126 (6)	0.0198 (6)	0.0121 (5)	0.0001 (5)	0.0002 (4)	−0.0003 (4)
C1A	0.0149 (7)	0.0250 (7)	0.0143 (6)	0.0015 (6)	−0.0001 (5)	−0.0010 (5)
C2A	0.0222 (8)	0.0326 (8)	0.0127 (6)	0.0034 (6)	−0.0010 (5)	−0.0031 (5)
C3A	0.0222 (8)	0.0296 (8)	0.0188 (6)	0.0041 (6)	−0.0022 (6)	−0.0113 (6)
C4A	0.0163 (7)	0.0193 (7)	0.0243 (7)	0.0013 (6)	0.0000 (6)	−0.0045 (5)
C5A	0.0106 (7)	0.0213 (7)	0.0161 (6)	0.0003 (5)	0.0007 (5)	−0.0018 (5)
C6A	0.0111 (7)	0.0212 (6)	0.0138 (6)	0.0004 (5)	0.0000 (5)	−0.0032 (5)
C7A	0.0146 (7)	0.0171 (6)	0.0134 (6)	0.0001 (5)	0.0004 (5)	−0.0015 (5)
C8A	0.0182 (7)	0.0181 (7)	0.0182 (6)	−0.0001 (6)	0.0002 (5)	−0.0009 (5)
C9A	0.0394 (10)	0.0153 (7)	0.0217 (7)	−0.0014 (6)	−0.0017 (6)	−0.0020 (5)
C10A	0.0134 (7)	0.0180 (6)	0.0150 (6)	−0.0003 (5)	0.0004 (5)	−0.0015 (5)
C11A	0.0177 (7)	0.0232 (7)	0.0114 (6)	−0.0008 (6)	−0.0001 (5)	0.0015 (5)
C12A	0.0207 (8)	0.0214 (7)	0.0227 (7)	−0.0001 (6)	0.0000 (6)	0.0042 (5)
C13A	0.0314 (9)	0.0275 (8)	0.0133 (6)	0.0002 (7)	−0.0006 (6)	0.0023 (5)
C14A	0.0229 (8)	0.0184 (7)	0.0192 (6)	−0.0008 (6)	0.0017 (6)	0.0004 (5)
O1B	0.0401 (7)	0.0182 (5)	0.0203 (5)	0.0010 (5)	−0.0003 (5)	0.0004 (4)
O2B	0.0405 (7)	0.0268 (5)	0.0115 (4)	0.0002 (5)	0.0009 (4)	−0.0036 (4)
O3B	0.0263 (6)	0.0182 (5)	0.0123 (4)	0.0005 (4)	0.0009 (4)	0.0017 (3)
N1B	0.0182 (6)	0.0150 (5)	0.0128 (5)	0.0010 (5)	0.0004 (4)	0.0006 (4)
N2B	0.0137 (6)	0.0188 (5)	0.0115 (5)	0.0001 (5)	0.0001 (4)	0.0007 (4)
C1B	0.0168 (7)	0.0214 (7)	0.0154 (6)	0.0011 (6)	−0.0002 (5)	0.0023 (5)
C2B	0.0219 (8)	0.0293 (8)	0.0115 (6)	0.0015 (6)	−0.0003 (5)	0.0024 (5)
C3B	0.0203 (8)	0.0308 (8)	0.0130 (6)	0.0021 (6)	−0.0006 (5)	−0.0061 (5)
C4B	0.0152 (7)	0.0190 (7)	0.0198 (6)	0.0013 (5)	−0.0008 (5)	−0.0043 (5)
C5B	0.0118 (7)	0.0183 (6)	0.0151 (6)	0.0011 (5)	−0.0004 (5)	−0.0001 (5)
C6B	0.0124 (7)	0.0184 (6)	0.0122 (6)	0.0008 (5)	−0.0001 (5)	−0.0011 (5)
C7B	0.0138 (7)	0.0182 (6)	0.0139 (6)	0.0011 (5)	0.0004 (5)	−0.0012 (5)
C8B	0.0175 (7)	0.0194 (7)	0.0181 (6)	0.0011 (6)	0.0006 (5)	−0.0026 (5)
C9B	0.0348 (9)	0.0198 (7)	0.0220 (7)	0.0030 (6)	−0.0005 (6)	−0.0069 (6)
C10B	0.0142 (7)	0.0209 (7)	0.0148 (6)	0.0007 (5)	−0.0001 (5)	−0.0006 (5)
C11B	0.0170 (7)	0.0257 (7)	0.0132 (6)	0.0003 (6)	−0.0002 (5)	0.0056 (5)
C12B	0.0212 (8)	0.0237 (7)	0.0238 (7)	0.0006 (6)	−0.0011 (6)	0.0060 (5)
C13B	0.0384 (10)	0.0216 (7)	0.0179 (6)	0.0014 (7)	0.0005 (6)	0.0058 (5)
C14B	0.0238 (8)	0.0160 (6)	0.0179 (6)	0.0005 (6)	0.0007 (5)	0.0002 (5)

### Geometric parameters (Å, °)

**Table:**

O1A—C8A	1.2463 (15)	O1B—C8B	1.2395 (16)
O2A—C10A	1.2123 (15)	O2B—C10B	1.2120 (15)
O3A—C10A	1.3419 (16)	O3B—C10B	1.3413 (16)
O3A—C11A	1.4514 (14)	O3B—C11B	1.4542 (15)
N1A—N2A	1.3040 (14)	N1B—N2B	1.3033 (14)
N1A—C6A	1.4132 (16)	N1B—C6B	1.4125 (15)
N1A—H1NA	0.904 (18)	N1B—H1NB	0.91 (2)
N2A—C7A	1.3287 (16)	N2B—C7B	1.3287 (16)
C1A—C2A	1.3905 (18)	C1B—C2B	1.3914 (18)
C1A—C6A	1.4126 (17)	C1B—C6B	1.4121 (17)
C1A—C13A	1.5095 (19)	C1B—C13B	1.5069 (19)
C2A—C3A	1.385 (2)	C2B—C3B	1.382 (2)
C2A—H2AA	0.930	C2B—H2BA	0.930
C3A—C4A	1.3845 (19)	C3B—C4B	1.3874 (18)
C3A—H3AA	0.930	C3B—H3BA	0.930
C4A—C5A	1.3979 (18)	C4B—C5B	1.3985 (17)
C4A—H4AA	0.930	C4B—H4BA	0.930
C5A—C6A	1.4060 (18)	C5B—C6B	1.4095 (18)
C5A—C14A	1.5112 (17)	C5B—C14B	1.5076 (17)
C7A—C8A	1.4690 (18)	C7B—C8B	1.4702 (18)
C7A—C10A	1.4876 (17)	C7B—C10B	1.4864 (17)
C8A—C9A	1.4953 (18)	C8B—C9B	1.4994 (18)
C9A—H9AA	0.960	C9B—H9BA	0.960
C9A—H9AB	0.960	C9B—H9BB	0.960
C9A—H9AC	0.960	C9B—H9BC	0.960
C11A—C12A	1.5075 (18)	C11B—C12B	1.5046 (19)
C11A—H11A	0.970	C11B—H11C	0.970
C11A—H11B	0.970	C11B—H11D	0.970
C12A—H12A	0.960	C12B—H12D	0.960
C12A—H12B	0.960	C12B—H12E	0.960
C12A—H12C	0.960	C12B—H12F	0.960
C13A—H13A	0.960	C13B—H13D	0.960
C13A—H13B	0.960	C13B—H13E	0.960
C13A—H13C	0.960	C13B—H13F	0.960
C14A—H14A	0.960	C14B—H14D	0.960
C14A—H14B	0.960	C14B—H14E	0.960
C14A—H14C	0.960	C14B—H14F	0.960
			
C10A—O3A—C11A	115.28 (9)	C10B—O3B—C11B	115.88 (10)
N2A—N1A—C6A	123.25 (11)	N2B—N1B—C6B	122.54 (11)
N2A—N1A—H1NA	116.5 (11)	N2B—N1B—H1NB	116.9 (12)
C6A—N1A—H1NA	120.3 (11)	C6B—N1B—H1NB	120.5 (12)
N1A—N2A—C7A	119.54 (11)	N1B—N2B—C7B	120.63 (11)
C2A—C1A—C6A	118.63 (13)	C2B—C1B—C6B	118.34 (12)
C2A—C1A—C13A	120.51 (12)	C2B—C1B—C13B	120.25 (12)
C6A—C1A—C13A	120.86 (12)	C6B—C1B—C13B	121.42 (12)
C3A—C2A—C1A	120.60 (13)	C3B—C2B—C1B	121.15 (12)
C3A—C2A—H2AA	119.7	C3B—C2B—H2BA	119.4
C1A—C2A—H2AA	119.7	C1B—C2B—H2BA	119.4
C4A—C3A—C2A	119.87 (12)	C2B—C3B—C4B	119.61 (12)
C4A—C3A—H3AA	120.1	C2B—C3B—H3BA	120.2
C2A—C3A—H3AA	120.1	C4B—C3B—H3BA	120.2
C3A—C4A—C5A	122.25 (13)	C3B—C4B—C5B	122.15 (12)
C3A—C4A—H4AA	118.9	C3B—C4B—H4BA	118.9
C5A—C4A—H4AA	118.9	C5B—C4B—H4BA	118.9
C4A—C5A—C6A	116.83 (12)	C4B—C5B—C6B	116.98 (11)
C4A—C5A—C14A	118.80 (12)	C4B—C5B—C14B	118.48 (11)
C6A—C5A—C14A	124.37 (11)	C6B—C5B—C14B	124.54 (11)
C5A—C6A—C1A	121.81 (11)	C5B—C6B—C1B	121.76 (11)
C5A—C6A—N1A	124.07 (11)	C5B—C6B—N1B	123.48 (11)
C1A—C6A—N1A	114.11 (12)	C1B—C6B—N1B	114.76 (11)
N2A—C7A—C8A	124.19 (11)	N2B—C7B—C8B	123.97 (11)
N2A—C7A—C10A	113.99 (11)	N2B—C7B—C10B	113.80 (11)
C8A—C7A—C10A	121.82 (11)	C8B—C7B—C10B	122.23 (11)
O1A—C8A—C7A	119.14 (11)	O1B—C8B—C7B	118.96 (11)
O1A—C8A—C9A	118.76 (12)	O1B—C8B—C9B	119.06 (12)
C7A—C8A—C9A	122.09 (11)	C7B—C8B—C9B	121.98 (12)
C8A—C9A—H9AA	109.5	C8B—C9B—H9BA	109.5
C8A—C9A—H9AB	109.5	C8B—C9B—H9BB	109.5
H9AA—C9A—H9AB	109.5	H9BA—C9B—H9BB	109.5
C8A—C9A—H9AC	109.5	C8B—C9B—H9BC	109.5
H9AA—C9A—H9AC	109.5	H9BA—C9B—H9BC	109.5
H9AB—C9A—H9AC	109.5	H9BB—C9B—H9BC	109.5
O2A—C10A—O3A	122.70 (11)	O2B—C10B—O3B	122.89 (12)
O2A—C10A—C7A	125.05 (12)	O2B—C10B—C7B	124.62 (12)
O3A—C10A—C7A	112.25 (10)	O3B—C10B—C7B	112.48 (10)
O3A—C11A—C12A	106.82 (10)	O3B—C11B—C12B	106.66 (10)
O3A—C11A—H11A	110.4	O3B—C11B—H11C	110.4
C12A—C11A—H11A	110.4	C12B—C11B—H11C	110.4
O3A—C11A—H11B	110.4	O3B—C11B—H11D	110.4
C12A—C11A—H11B	110.4	C12B—C11B—H11D	110.4
H11A—C11A—H11B	108.6	H11C—C11B—H11D	108.6
C11A—C12A—H12A	109.5	C11B—C12B—H12D	109.5
C11A—C12A—H12B	109.5	C11B—C12B—H12E	109.5
H12A—C12A—H12B	109.5	H12D—C12B—H12E	109.5
C11A—C12A—H12C	109.5	C11B—C12B—H12F	109.5
H12A—C12A—H12C	109.5	H12D—C12B—H12F	109.5
H12B—C12A—H12C	109.5	H12E—C12B—H12F	109.5
C1A—C13A—H13A	109.5	C1B—C13B—H13D	109.5
C1A—C13A—H13B	109.5	C1B—C13B—H13E	109.5
H13A—C13A—H13B	109.5	H13D—C13B—H13E	109.5
C1A—C13A—H13C	109.5	C1B—C13B—H13F	109.5
H13A—C13A—H13C	109.5	H13D—C13B—H13F	109.5
H13B—C13A—H13C	109.5	H13E—C13B—H13F	109.5
C5A—C14A—H14A	109.5	C5B—C14B—H14D	109.5
C5A—C14A—H14B	109.5	C5B—C14B—H14E	109.5
H14A—C14A—H14B	109.5	H14D—C14B—H14E	109.5
C5A—C14A—H14C	109.5	C5B—C14B—H14F	109.5
H14A—C14A—H14C	109.5	H14D—C14B—H14F	109.5
H14B—C14A—H14C	109.5	H14E—C14B—H14F	109.5
			
C6A—N1A—N2A—C7A	179.03 (12)	C6B—N1B—N2B—C7B	−179.54 (12)
C6A—C1A—C2A—C3A	−0.2 (2)	C6B—C1B—C2B—C3B	−0.2 (2)
C13A—C1A—C2A—C3A	179.61 (14)	C13B—C1B—C2B—C3B	179.80 (14)
C1A—C2A—C3A—C4A	1.1 (2)	C1B—C2B—C3B—C4B	−0.2 (2)
C2A—C3A—C4A—C5A	−0.8 (2)	C2B—C3B—C4B—C5B	0.1 (2)
C3A—C4A—C5A—C6A	−0.3 (2)	C3B—C4B—C5B—C6B	0.3 (2)
C3A—C4A—C5A—C14A	179.28 (13)	C3B—C4B—C5B—C14B	−179.27 (13)
C4A—C5A—C6A—C1A	1.2 (2)	C4B—C5B—C6B—C1B	−0.78 (19)
C14A—C5A—C6A—C1A	−178.33 (13)	C14B—C5B—C6B—C1B	178.79 (13)
C4A—C5A—C6A—N1A	−178.53 (12)	C4B—C5B—C6B—N1B	179.62 (12)
C14A—C5A—C6A—N1A	1.9 (2)	C14B—C5B—C6B—N1B	−0.8 (2)
C2A—C1A—C6A—C5A	−1.0 (2)	C2B—C1B—C6B—C5B	0.7 (2)
C13A—C1A—C6A—C5A	179.22 (13)	C13B—C1B—C6B—C5B	−179.29 (13)
C2A—C1A—C6A—N1A	178.77 (12)	C2B—C1B—C6B—N1B	−179.62 (12)
C13A—C1A—C6A—N1A	−1.02 (19)	C13B—C1B—C6B—N1B	0.34 (19)
N2A—N1A—C6A—C5A	2.0 (2)	N2B—N1B—C6B—C5B	−2.4 (2)
N2A—N1A—C6A—C1A	−177.74 (12)	N2B—N1B—C6B—C1B	178.02 (12)
N1A—N2A—C7A—C8A	−0.6 (2)	N1B—N2B—C7B—C8B	0.9 (2)
N1A—N2A—C7A—C10A	179.99 (11)	N1B—N2B—C7B—C10B	−179.37 (11)
N2A—C7A—C8A—O1A	−0.6 (2)	N2B—C7B—C8B—O1B	−1.9 (2)
C10A—C7A—C8A—O1A	178.82 (13)	C10B—C7B—C8B—O1B	178.34 (13)
N2A—C7A—C8A—C9A	−179.80 (13)	N2B—C7B—C8B—C9B	177.42 (13)
C10A—C7A—C8A—C9A	−0.4 (2)	C10B—C7B—C8B—C9B	−2.3 (2)
C11A—O3A—C10A—O2A	0.89 (19)	C11B—O3B—C10B—O2B	−1.24 (19)
C11A—O3A—C10A—C7A	−178.87 (11)	C11B—O3B—C10B—C7B	178.20 (11)
N2A—C7A—C10A—O2A	178.94 (13)	N2B—C7B—C10B—O2B	178.42 (13)
C8A—C7A—C10A—O2A	−0.5 (2)	C8B—C7B—C10B—O2B	−1.8 (2)
N2A—C7A—C10A—O3A	−1.31 (16)	N2B—C7B—C10B—O3B	−1.00 (17)
C8A—C7A—C10A—O3A	179.24 (12)	C8B—C7B—C10B—O3B	178.78 (12)
C10A—O3A—C11A—C12A	179.37 (11)	C10B—O3B—C11B—C12B	−177.50 (11)

### Hydrogen-bond geometry (Å, °)

**Table:**

*D*—H···*A*	*D*—H	H···*A*	*D*···*A*	*D*—H···*A*
C2B—H2BA···O2B^i^	0.93	2.54	3.2442 (16)	133
N1A—H1NA···O1A	0.904 (18)	1.790 (18)	2.5366 (14)	138.3 (15)
N1B—H1NB···O1B	0.91 (2)	1.81 (2)	2.5512 (15)	136.2 (16)
C11B—H11C···Cg1^ii^	0.96	2.68	3.5516 (15)	150
C11B—H11D···Cg1^iii^	0.96	2.60	3.4832 (15)	152
C11A—H11A···Cg2^iv^	0.96	2.61	3.4633 (15)	147
C11A—H11B···Cg2^v^	0.96	2.65	3.5304 (15)	151

Symmetry codes: (i) *x*, −*y*+1/2, *z*+1/2; (ii) −*x*, *y*−1/2, −*z*+1/2; (iii) −*x*+1, *y*−1/2, −*z*+1/2; (iv) −*x*, *y*+1/2, −*z*+1/2; (v) −*x*+1, *y*+1/2, −*z*+1/2.
